# Effect of inherent misalignment and head motion in neurological PET/MR with the Philips Ingenuity TF – phantom and patient study

**DOI:** 10.1186/2197-7364-1-S1-A65

**Published:** 2014-07-29

**Authors:** Jarmo Teuho, Jarkko Johansson, Virva Saunavaara, Nina Kemppainen, Mika Teräs

**Affiliations:** Turku PET Centre, Turku University Hospital, Turku, Finland

The aim of the study was to evaluate the effect of misalignment and head motion on image quantification in PET/MR with a novel brain phantom and a healthy control group. The phantom was imaged at two time points in PET/MR, concurrently with PET and PET/CT. Phantom images were evaluated visually and the relative difference in hemispheric accumulation was calculated. Difference in cortical accumulation in a healthy control group was evaluated from non-attenuation corrected (NAC) and MR attenuation corrected (MRAC) images. Regional ROI mean values from F^18^-FDG ratio images and regional hemispheric asymmetries were calculated. Controls were divided to high and low asymmetry groups. A student’s t-test (p<0.005) for group difference and NAC versus MRAC data was performed. Finally, mean PET-MR registration parameters were measured. Only the first phantom scan exhibited asymmetry in lateral frontal cortex (17%) and temporal cortex (19%). Correcting the misalignment of 2.63mm reduced the asymmetry to less than 5%, to a level seen in PET and PET/CT. A significant asymmetry was found in the temporal and parietal cortex between groups in MRAC data with no significant asymmetry in NAC data. Asymmetries in affected MRAC data in temporal and parietal cortex were 9.4% and 11.7%. NAC data from both groups had asymmetry less than 5% in all regions. Both groups had significant y- and z-translation, while only the asymmetry group had significant z-rotation and x-translation. The shift in x-, y-, or z-direction in both groups was less than 4 mm, with no significant differences. Thus, PET-MRAC misalignment may cause under- and overestimation of attenuation in the lines of response on opposite sides of the cortical regions, resulting to asymmetric difference between the hemispheres. Our findings stress the need for novel QC procedures for PET-MR alignment and suggest confirming the quality of PET-MRAC alignment from PET-NAC images.

